# A contextual-compositional approach to discover associations between health determinants and health indicators for neonatal mortality rate monitoring in situations of anomalies

**DOI:** 10.1371/journal.pone.0310413

**Published:** 2024-12-11

**Authors:** Laís Baroni, Lucas Scoralick, Augusto Reis, Kele Belloze, Marcel Pedroso, Ronaldo Alves, Cristiano Boccolini, Patricia Boccolini, Eduardo Ogasawara

**Affiliations:** 1 Federal Center for Technological Education of Rio de Janeiro (CEFET/RJ), Rio de Janeiro, RJ, Brazil; 2 Oswaldo Cruz Foundation (Fiocruz), Rio de Janeiro, RJ, Brazil; 3 Petrópolis Medical School, Petrópolis, RJ, Brazil; University of Sao Paulo: Universidade de Sao Paulo, BRAZIL

## Abstract

**Introduction:**

Epidemiology is considered both a field of research and a methodological approach within the broader health sciences. It aims to understand health-related events’ causes and effects and provide the evidence necessary to prevent disease and implement effective control and prevention strategies. One of the main focuses of epidemiology is identifying the determinant factors in the health situation of populations since health-related anomalies are not randomly distributed among people. This understanding brings up the necessity of considering each place’s particularities and observing the regularity of diseases in a population context.

**Methods:**

We present the Contextual-Compositional Approach (CCA) for the discovery of associations between Health Indicators (HI) and Health Determinants (HD) for neonatal mortality rate monitoring in situations of anomalies. CCA uses time series concepts, anomaly detection, and data distribution between classes for studying HD under expected conditions and comparing them to the anomaly conditions indicated by the anomaly detection in the HI. CCA is evaluated using a neonatal mortality database in health facilities in Rio de Janeiro, Brazil.

**Results:**

The results show that CCA can reveal essential associations between the health condition and the population’s social, economic, and cultural characteristics on different scales.

**Conclusion:**

CCA stands out because it is easy to apply and understand, requiring little computational resources and parameters.

## Introduction

Epidemiology is essential in public health because it studies the health-disease-care process in human populations [1]. One of the main focuses of this field is to identify the determinant factors in the health situation of populations once it is understood that health-related anomalies are not randomly distributed among people [2, 3]. This understanding brings up the necessity of considering each place’s particularities and observing the distribution and regularity of diseases in a population context [4, 5]. The particularities of each place can be represented by Health Determinants (HD). The HD are variables (social, economic, and cultural) that are related to the health situation of the population in that place. In contrast, the regularity of diseases can be evidenced by the Health Indicators (HI). The HI are variables that measure the state (in a sectional cut) and changes (in a longitudinal cut) of the population health situation.

HD and HI can be presented by time series. They are presented as a collection of observations that are regularly collected over time. In a time series analysis, it is frequently possible to observe a significant change in the behavior of a time series occurring in a determined time or time interval [6]. Such behavior change usually characterizes the occurrence of an anomaly [7, 8]. A detected anomaly in a time series of HI may represent a phenomenon with specific meaning and defined in a determined knowledge domain [9].

Studies that seek to recognize behavioral patterns of HD in different Study Environment (SE) are essential for understanding how these HD may or may not influence the health of the people [10, 11]. In that context, there are two possible approaches to SE: compositional and contextual [12]. The compositional approach takes into consideration different individual characteristics of people who attend each SE, represented by HD (for example, the average age of the population). The contextual approach concerns the characteristics of that area’s physical and social environment. The compositional and contextual characteristics create a dichotomy in the search for relations between HI and HD in different SE.

An example of this dichotomy comprehends an individual with high acquisitive power who enjoys the best health conditions despite living in a low-class neighborhood. In this case, studying the individuals seems more promising (compositional approach). On the other hand, one cannot neglect the influence that this environment has on this individual. Regardless of the individual, environmental conditions like sanitation or pollution interfere with the health of everyone who frequents that environment, despite their financial status (contextual approach) [13, 14].

The compositional and contextual effects do not apply only when the focus is on context as geographic space but also when the context is viewed in administrative, temporal, or institutional terms. In institutional terms, the variations in the performance of health service activities between different providing units can be attributed to the type of client that the particular units serve (compositional) and also to the nature of the environment in which the service is provided (contextual) [15]. That is a common situation, especially in continental countries like Brazil, which presents expressive historical and social inequalities that reflect on the population’s health [16, 17]. In that regard, evidence of health inequalities must be considered based on any research initiative or action focusing on interpretation and intervention to improve health. Disregarding these inequalities may hide substantial differences between population subgroups and cause bias in health research results. Therefore, the research question of this article consists of studying the compositional effects of the SE without being covered or influenced by contextual effects.

In this paper, we present the Contextual-Compositional Approach (CCA), which aims to reveal associations between HD and HI for neonatal mortality rate in SE. The proposed method uses descriptive statistics and anomaly detection in time series. CCA can consider different contextual effects present in SE and returning interpretable associations. At the same time, the anomaly detection methods identify possible changes, whether compositional or contextual, that occur naturally with time.

To the best of the authors’ knowledge, CCA is the first approach to generally discover contextual and compositional associations in SE through time series anomaly detection applied in health research [18–20]. As presented, contextual and compositional are critical to determining health disease, and a time series analysis can detect changes in the characteristics over time. All this should improve anomaly detection once we consider more dimensions for the problem.

## Materials and methods

Contextual-Compositional Approach (CCA) aims to analyze compositional and contextual characteristics of SE. The method consists of comparing HD in typical conditions and under detected anomalous conditions [18, 20] in the time series of HI. Considering the contextual effects, the pattern is defined at SE level (health facility, for example).

**Fig 1:**
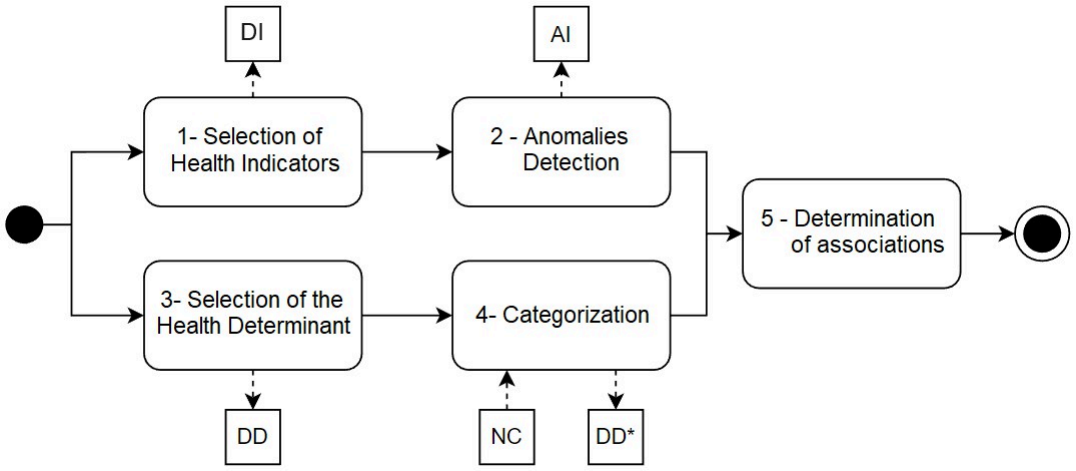
CCA association discovery process. The activities flow starts from the left, with the black ball, then terminates at the right, with the circled black ball. Each rounded square represents an activity referenced as a subsection of this research. The tiny squares represent the datasets: Dataset of Indicators (ID); Anomalies of Indicators (AI); Dataset of Determinants (DD); Number of Classes (NC); Dataset of Determinants modified by categorization (DD*).

To understand the proposed goals, CCA is divided into five parts, as pictured in the activity diagram of Fig 1. The process starts with the selection of HI and HD represented by the Dataset of Indicators (DI) and Dataset of Determinants (DD) datasets, respectively. The anomaly detection is applied in DI dataset, generating a Set of Interest (SoI) and Anomalies of the Indicators (AI). DD goes through categorization receiving the Number of Classes (NC) parameter and generating a dataset DD*, derived from DI. Thereby, it is considered the SoI for AI to determinate a sample of DD*. From the analysis of this sample, the associations are identified.

### Selection of the health indicators

The health indicators (HI) are summary measures used to express the health conditions of a population, as well as the performance of the health system. It can be used as a guiding parameter for decision-making regarding management and strategic planning actions [21]. Some very common indicators in epidemiology are death and birth rates, incidence of infectious diseases, and prevalence.

It is common for indicators to be represented through time series. From the time series, it is possible to describe and monitor the health situation of a population using regular observation of the phenomenon over time. This feature is important for comparing temporal data and analyzing tendencies and predictions.

Formally, a time series *X* is a sequence of observations over time < *x*_1_, *x*_2_, …, *x*_*n*_ >, where *n* is the number of observations. Consider a time series dataset *DX* as a collection of *m* time series *X*. Specific observations of this dataset are presented by *dx*_*i*,*j*_, indexed in the time *i* ∈ {1, …, *n*} and in the series *j* ∈ {1, …, *m*}.

HI are presented by the DI (time series dataset). DI is composed of *m* time series of size *n*, one for each SE to be considered. Therefore, the observation *di*_*i*,*j*_ represents the HI value at time *i* and SE *j*. The SE should share the same nature, including counties, census sectors, and healthcare facilities. In other words, it must have the same set of attributes.

### Anomalies detection

Time series anomalies are single observations or sequences of observations where the series has altered behavior. More specifically, anomalies are observations that seem to have been generated by a process different from the one that generated the entire time series. Since anomalies stand out in a time series, they can be modeled based on distance functions from expected observations [22].

Several methods aim to detect anomalies using model deviation analysis based on classification, grouping, or statistical techniques [18, 23, 24]. CCA adopts Forward and Backward Inertial Anomaly Detector (FBIAD) [25] since it is based on the concept of inertia. The concept of inertia in time series is related to the idea that a force exists to keep the observations in a stable state over time [26]. Inertia remains until a rupture occurs. The FBIAD method is chosen because it does not quickly react to short-term phenomena and avoids spurious disturbances. Moreover, regarding the implementation of CCA, this feature is important to ensure the vast applicability of the method, whatever the health determinant or its behavior in the SE.

Therefore, in this step, each time series of the dataset DI is submitted to the FBIAD method to detect anomalies [25]. All the detected anomalies for each time series of DI are stored in a AI set. Formally, the AI = {[*i*_1_,*j*_1_], ⋯, [*i*_*o*_,*j*_*o*_]}, where the size of the set is *o* (|AI| = *o*). The set AI represents, in the context of this work, the SoI for the association between anomalous occurrences in HI and the behavior of HD.

### Selection of the health determinants

The health determinants (HD) are the conditions that influence the occurrence of health problems and their risk factors in the population [27]. It includes work, basic sanitation, and economic, cultural, biological, environmental, and behavioral factors. A challenge related to HD is being able to assess the influence that each one of them has on the health status of the population.

In the context of inequalities, the HD relate the compositional effects of each group of individuals to each SE. In addition, considering the peculiar characteristics of each subgroup allows highlighting the most disadvantaged in the context of each SE and not in the general context. It is an efficient strategy to reinforce the relationship between unfavorable conditions and unwanted anomalies in HI.

Therefore, for this study, the HD must be variables that characterize a favorable condition for the occurrence of the disease. For example, it is believed that an individual’s age (HD) is related to the incidence of Alzheimer’s disease (HI). However, the favorable conditions for the occurrence of the disease, in this case, would be advanced-age individuals rather than the fluctuation of individuals ages themselves. In this case, the appropriate HI is the variation in the percentage of elderly patients in SE, which could be a hospital. The percentage is used to aggregate the individuality within the HD since the compositional effects deal with the particularities of the group: the one who composes or attends the SE.

In this way, an interval of interest is defined within the variable, and the search for the relationship is more targeted, focusing on the risk factor. This activity requires a modeling of the HD. In epidemiology, it is common to use absolute values, percentage variation, and variable rates. The sensitivity to establishing critical types and intervals of favorable conditions for the disease is crucial for the efficiency of the proposed method.

HD are represented by the DD (time series dataset). The DD is a *m* time series collection. Each time series corresponds to a SE. One specific observation of this dataset is represented by *dd*_*i*,*j*_, indexed in time *i* (*i* ∈ {1, ⋯, *n*}) and in series *j* (*j* ∈ {1, ⋯, *m*}). The health determinant is the DD dataset in *m* time series of size *n*. The observation *dd*_*i*,*j*_ is the value of the health determinant at time *i* and at SE *j*.

Note that the number *m* of time series in the DD dataset must be the same as in the DI dataset since they are the same SE that must be compared in the study. Likewise, the sampling period and sampling interval must be equal: the number of observations *m* and the interval it varies within the same period as in the series in DD. Thus, the observation *di*_*i*,*j*_ from DI dataset and observation *dd*_*i*,*j*_ from DD dataset bring, respectively, information about the health indicator and the health determinant in the same SE and time.

### Categorization

Contextual-Compositional Approach (CCA) transforms HD into categorical values. For this purpose, the HD are normalized in classes using quantiles. Classification by quantiles seeks to allocate the same number of observations per class, regardless of the amplitude of each class. For that, it is necessary to define limits that indicate which position a specific value is concerning the other historical data, making a classification of interest. The process begins with determining the number of classes (NC) to be considered. The choice of the value of NC is associated with the desired generalization, and the greater the NC, the smaller the generalization since less data is considered in each class. It can be chosen arbitrarily, considering the study data and the desired generalization.

The size *t* of each class can be defined accordingly with the series size to be categorized using the formula: $t=\frac{m}{NC}$. Therefore, the categorization considers for the q-th class, such that 1 ≤ *q* ≤ *NC*, the comprehended values between the positions *t* × (*q* − 1) + 1 to *q* × *t* of the series, arranged in ascending order. As an example, a time series of HD in a SE with 100 observations in time and NC = 4. The 25 lowest observed values are labeled as class 1. This process continues until they form four classes, each with 25 elements.

Once the NC is determined, each series *Y* of the dataset DD is submitted to the categorization process. This way, the dataset DD is transformed into another dataset, called DD*, which retains the same characteristics, but instead of presenting the observations *dd*_*i*,*j*_ as literal values of HD, it presents the observation $y_{i,j}^{*}$ with the indication of the class that it belongs.

Through quantile classification on the HD series in each SE, it is guaranteed that what is in each category is supported by the variation of the data in SE. Therefore, it is possible to observe the information within the context of that SE and not the general context. This property is important due to the heterogeneity that can occur in the behavior of HI in different SE, meeting the understanding of the heterogeneity of social and geographic conditions of the general environment that covers all SE considered. That way, for each SE, the cutoff values between categories are different. This point is crucial to meet the proposal of individual consideration and mitigation of contextual influences. Furthermore, sorting by quantile mitigates the influence of possible anomalies in the data distribution.

### Determination of associations

Associations can be analyzed once the HI events have been identified and the HD are categorized. The values of the dataset DD* are considered a Set of Interest (SoI) only in cases (place and date) identified in Anomalies of the Indicators (AI); in other words, those that were considered anomalous in HI. SoI=DD*∣[i,j]∈AI. So, SoI is a sample of DD*.

The analysis of the associations between HI and HD is based on the Central Limit Theorem and statistical inference, which addresses the characteristics of a random sample and its ability to represent the population [28]. If a sample, accordingly, demonstrates a distribution different from its original population, it implies that the sampling process was not conducted randomly but exhibits some bias.

It is precisely this idea that reveals the associations. If there is no relationship between HI and HD, it would be like extracting a random sample from HI. Thus, the number of occurrences in each class would remain equivalent (approximately 25% data in each class, if NC = 4). Once an imbalance occurs in the class distribution, it is considered that the sampling was not random. Therefore, it is possible to relate the behavior of HD with that of HI.

To ensure that the distribution between classes differs from expected, we used Student’s t-test with a confidence level of 95% (p-value of 0.05). For each SE, the occurrences in each class are counted. For example, in a specific SE, there are ten anomalies found in its HI. In this set of interests of size ten, the HD with three classes to be analyzed shows one value in class one, two values in class two, and seven in class three. These values are transformed in percentages so that class one has 10% of the data, in class two, 20%, and in class three, 70%. It is done for each SE, creating a set of percentages for all SE for each class. The t-test is then conducted to compare the mean of these sets with the expected mean for each class, in this case, 33.33%.

When the null hypothesis is rejected, this category has more or less data than expected if there is no relationship between HD and anomalies in HI. These are the HD related to the anomalous behavior of the HI. It is still possible to understand how this relationship occurs by observing which class shows a significantly higher or lower frequency than expected.

### Neonatal mortality rate dataset

We used a machine-readable and open-access database from the Assessing the Impact of Hospital-Based Breastfeeding Interventions on Infant Health project. Detailed information on the project’ database is available elsewhere [29, 30]. In summary, it gathers official data from the Mortality Information System (SIM), Live Birth Information System (SINASC), and the National Registry of Healthcare Facilities (CNES), obtained via official and public sources from the Brazilian Ministry of Health. The database includes epidemiological, sociodemographic, and breastfeeding technologies data aggregated by months/years (from August 2005 to December 2017) and healthcare facilities in Brazil.

For the application and validation of CCA, we used a subset of this database regarding 65 health facilities in the metropolitan area of Rio de Janeiro city from January 2006 to December 2017, which met two eligibility criteria: (i) 100 or more births per year and at least one obstetric bed; (ii) agreement with the Brazilian Unified Health System (SUS) at any time in the historical series. HI selected was the Neonatal Mortality Rate (NMR), defined by the number of deaths of children aged 0 to 27 complete days of life per thousand live births. Additionally, four attributes were considered HD: weeks of gestation, attended prenatal consultations, type of delivery, and mother’s education. The decision to use this dataset was due to the challenge it brings by presenting variant patterns in the behavior of the time series between the different Study Environments (SE).

**Fig 2:**
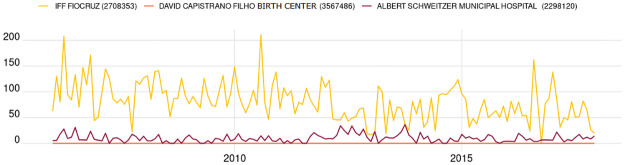
Neonatal mortality rate in three health facilities in the metropolitan region of Rio de Janeiro.

To illustrate this challenge, Fig 2 is presented, which shows the neonatal mortality rate, our HI, in three health facilities.

In Fig 2, the David Capristano Filho Birth Center has a Neonatal Mortality Rate (NMR) equal to zero throughout the period. A possible explanation for this is the fact that this maternity only receives low-risk pregnant women, pregnant with a single baby, who do not have chronic diseases or develop complicating factors such as diabetes and hypertension. At the other extreme, there is the Fernandes Figueira Institute (IFF), which, in turn, only receives pregnant women in need of specialized and more complex assistance. Consequently, the NMR is significantly higher than other health facilities. These two health facilities have a particular behavior, given their specificities of care or structure. The Albert Schweitzer Municipal Hospital presents a more standard behavior, where its NMR has a mean and variation similar to most health facilities in the study.

This characteristic highlights the importance of considering health facilities on a case-by-case basis, including when defining the occurrence of anomalies in the NMR. For example, if a default value were defined, the entire IFF series could be considered an anomaly, which is not convenient in the proposed approach.

The CCA was implemented in R. The anomaly detection was done using Harbinger [24]. All code and data are made available at https://eic.cefet-rj.br/~dal/cca. The data utilized for this research are available in RData format at the git public repository at https://github.com/cefet-rj-dal/cca/blob/main/df.RData.

## Results

### Selection of the health indicators and anomaly detection

**Fig 3:**
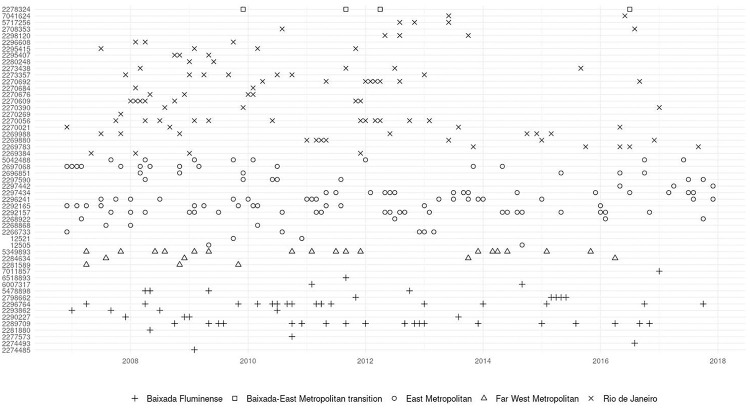
Occurrence of anomalies in the neonatal mortality rates of the health facilities in the study.

We used the Neonatal Mortality Rate (NMR) as health indicators (HI) for the experimental evaluation. In the Contextual-Compositional Approach (CCA), the only parameter required to perform the anomaly detection is the window size, defined as 12 (one year), respecting the monthly sampling in which the data are presented. There were 304 anomalies in 120 dates (month/year) and 53 of 65 health facilities. Fig 3 summarizes the anomalies, spatially and temporally. By analyzing Fig 3, an obvious pattern is not perceptible in anomalies, which are well distributed in space and different parts of the metropolitan region of Rio de Janeiro. Note, however, that although the average is 4.7 anomalies per maternity, some health facilities have frequent occurrences of anomalies, for example, Clissil São Silvestre Clinic (CNES 2292157), with 22 anomalies.

This observation of the varied distribution of anomalies in time and space shows no obvious relationship between time, space, and anomalies in NMR, bringing insights to the proposed approach.

### Selection of the health determinants and categorization

For a better presentation of the Contextual-Compositional Approach (CCA) results, it was decided to test it to determine the association of HI with four different HD: type of delivery, mother’s education, number of prenatal visits and weeks of gestation. Considering the data presentation in the utilized dataset, some manipulations were necessary to define the HD to meet the requirements demanded by the approach, as described in Section Selection of the health determinants. Table 1 presents a summary justified in the next paragraphs.

**Table 1:** Origin and interpretation of HD.

Health Determinant	Source attribute	Interpretation
Prematurity	Gestation Weeks	Percentage of births occurring before the 37th week of gestation
Inadequate prenatal care	Attended prenatal appointments	percentage of mothers who attended less than six consultations before giving birth
Cesarean deliveries	Type of delivery	Percentage of mothers who underwent cesarean delivery
Mothers with low education	Mother’s education	Percentage of illiterate mothers or with incomplete primary education

For weeks of gestation, the HD corresponds to the proportion of deliveries performed before the 37º week of gestation, that is, cases of prematurity. Regarding the number of prenatal visits, the HD corresponds to the percentages of mothers who did not attend at least six prenatal visits before delivery, the minimum number recommended by the Ministry of Health [31].

For the attribute indicative of delivery (cesarean or vaginal), the percentage of cesarean delivery was chosen as HD since it is a surgical procedure, not a natural one. Lastly, the mother’s education is treated as a social determinant. That way, HD corresponds to the percentage of mothers with up to seven years of schooling. It corresponds to the percentage of mothers who are illiterate or have incomplete primary education.

A different number of classes (NC) for categorization was evaluated. It was organized into two, three, four, and five classes. The objective is to enrich the experimental evaluation and expand the possibilities for discussing the results.

### Determination of associations

Table 2 showcases the results for the studied HD in four class intervals: from two to five. For these intervals, it is expected to have 50%, 33.33%, 25%, and 20% of the data in each class. The highlighted values comprise the cases in which the t-test rejected the hypothesis that the presented averages are equal to the expected ones. That is, these are the HD that are related to the HI (NMR). Observing which classes the associations occur in and if the averages are higher or lower than expected makes it possible to interpret how this association occurs.

**Table 2:** Results for the HD of the study.

	two classes (50% in each)		three classes (33,33% in each)		
	**1**	**2**	**1**	**2**	**3**
**Mothers with low education**	42.49%	57.51%	28.90%	30.97%	40.14%
**Cesarean deliveries**	43.84%	56.16%	32.87%	28.62%	38.51%
**Inadequate prenatal care**	42.57%	57.43%	27.58%	33.63%	38.79%
**Prematurity**	**41.40%**	**58.60%**	**25.34%**	34.63%	40.03%
	four classes (25% in each)				
	**1**	**2**	**3**	**4**	
**Mothers with low education**	19.72%	22.77%	25.39%	32.12%	
**Cesarean deliveries**	29.09%	**14.83%**	27.67%	28.41%	
**Inadequate prenatal care**	22.38%	21.13%	29.16%	27.33%	
**Prematurity**	**17.01%**	24.47%	25.95%	32.57%	
	five classes (20% in each)				
	**1**	**2**	**3**	**4**	**5**
**Mothers with low education**	15.42%	16.87%	19.61%	25.11%	22.99%
**Cesarean deliveries**	21.50%	**13.51%**	19.55%	22.52%	22.91%
**Inadequate prenatal care**	21.58%	**11.94%**	18.64%	25.81%	22.03%
**Prematurity**	**11.94%**	19.31%	19.12%	21.78%	**27.85%**

The highlighted values are those in which the t-test refuted the hypothesis of mean equal to expected.

Regarding the HD of prematurity, it was observed that anomalies occurred more in higher categories. That is, the increase in the NMR is related to the increase in the birth of premature babies. In the division into three and four classes, only an association is observed with the decrease in the lowest class (lowest risk), meaning fewer anomalies occur when fewer premature infants exist. In the division into five classes, in addition to the above observation, it is also revealed that there is an increase in the NMR when many premature babies are born.

### Evaluation

The analysis of associations between health indicators (HI) and health determinants (HD) is based on the fact that a random sample can represent the population with a certain degree of fidelity. So, the unbalanced distributions between the classes in the SoI of an HD represent that that HD is related to the anomalies in the HI. It is because if HD had no relationship with HI or if the sample used from HD was random, in other terms, it did not consider the SoI according to the anomalies of HI, the frequencies between classes would remain balanced.

Therefore, this subsection shows the results of tests that seek to corroborate the assumption mentioned earlier. Two test configurations are made for HD with three categories:

1. a random HD is created (with equal distribution between the classes), and the t-test is carried out within the SoI and,
2. a HD of the study is used in random samples of the same size as the SoI.

Each configuration went through 1,000 rounds, changing the random set (of HD in configuration one and samples in configuration 2) for each number of classes considered.

Test results in the first configuration show that only 6.20%, 4.70%, and 5.60% of tests in classes 1, 2, and 3, respectively, refuted the null hypothesis. For the second configuration, HD “number of births” was used. The results showed that 6.30%, 5.40%, and 5.70% tests in classes 1, 2, and 3 refute the null hypothesis.

In most cases, the averages shown are not considered different than expected. This evaluation is based on the veracity of the hypothesis. It highlights that when divergences are found in the HD, they are relevant and interesting to investigate.

## Discussion

In this article, we propose the Contextual-Compositional Approach (CCA), which is capable of revealing associations between health indicators (HI) and health determinants (HD) for neonatal mortality rate (NMR), taking into account the contextual characteristics of each unit of Study Environments (SE). It reduces the bias that can be caused when the units of analysis are inserted in different contexts and make it difficult to compare them. There is a need to adjust the data for the use of CCA and interpret the results found. It is necessary that there are time series of HI and HD in each SE and that they have the same frequency. It should be noted that these requirements are easy to obtain. Moreover, health datasets commonly present these variable formats or composition possibilities.

For the analysis, the HI of NMR and HD related to the mother’s situation and childbirth were used. Neonatal mortality, defined as the number of deaths of children between 0 and 27 full days of life, represents a global public health problem. Although infant mortality rates have been decreasing in Brazil and worldwide, the decrease in NMR occurs at a slower pace than that observed in infant mortality, to the point that, currently, this component corresponds to about two-thirds of the total number of infant deaths. These indicators reflect, in general, the mother’s socioeconomic and health conditions, as well as prenatal, delivery, and newborn care [32]. In this way, infant mortality is not only an indicator of child health but an indicator of the living conditions of a population [33].

In analyzing time series data, anomalies detected in the NMR may represent relevant phenomena in a given knowledge domain [9]. More specifically, they represent undesirable events such as local outbreaks (e.g., hospital-acquired infections) or large-scale outbreaks (e.g., epidemics). They may also reflect problems in the facility, whether administrative, financial, or political. Understanding the patterns common to these anomalies is pertinent to avoiding such situations or, at least, predicting their occurrence.

About the determination of associations, in addition to being easy to interpret, the result is in line with the results of several other studies carried out in the same context [34–36].

Regarding the number of classes used in the analysis, we can see that the two relevant HD were revealed in all class divisions. However, when we use more classes, some new relationships are revealed. Furthermore, for the HD of cesarean deliveries and the number of prenatal consultations, associations appear only in the 4th and 5th class intervals. Associations appear in the second class in both HD, but interpreting these occurrences is not trivial. CCA showed that premature births were associated with neonatal mortality at statistically significant levels since Student’s t-test shows that results for this determinant are statistically different than random results, especially for the higher classes (three class: 25.34%, when random is 33.33%; four classes: 17.01%, when random is 25%; five classes: 11.94%, when random is 20%). Similarly, no relationship was found between the mother’s education level and the increase in the NMR. This observation has already been made in previous research [34, 37, 38]. Furthermore, a weaker relationship was observed with the occurrence of cesarean deliveries and prenatal care. An important characteristic is the possibility of observing the results in different numbers of classes, making the analysis more or less discretized.

### Limitations

Among the limitations of CCA, it is possible to mention that it depends on the correct preparation of the data, which can be a challenge depending on the availability and quality of the datasets. The quantity and quality of data directly influence the validity of the analyses, and it is crucial to have an adequate number of observations and anomalies. Not considering complex interrelationships between HDs may lead to limited conclusions, suggesting that more sophisticated analyses could be needed to capture the full complexity of the relationships. The results should be interpreted carefully, given that statistically significant associations do not imply causality. We expect to evaluate the method for other datasets in different epidemiological contexts for future contributions.

## Conclusion

The method proposed in this article proved useful in discovering relationships between HI and HD, which can be shown by running it on neonatal mortality rate dataset. The hypothesis on which it is based turned out to be coherent. The method stands out because it is easy to apply and understand, requiring little computational resources and parameters.

Still, for a good execution of the method, it is necessary to have a sufficient amount of SE and observations in the time series to enable analysis of samples that have statistical validity. It also depends on the amount of anomalies found in HI. The method discovers the anomalies and evaluates the HD classes within the context of each SE. Besides, the CCA is limited to study the relationship between the HD and HI. However, it is reasonable to have other relationships that can influence each other, such as, for example, the occurrence of premature birth with the need to perform a cesarean section. Finally, it is also important to emphasize that although CCA reveals associations between HI and HD with statistical significance, it is not possible to affirm the causal relationship between them.
